# The role and signal pathways of opsin 3 in the skin: from light perception to pathophysiological mechanisms

**DOI:** 10.3389/fmed.2026.1857820

**Published:** 2026-07-15

**Authors:** Qian Zhang, Yanyan Feng

**Affiliations:** Department of Dermatology, Sichuan University affiliated Chengdu Second People’s Hospital, Chengdu Second People’s Hospital, West China School of Medicine, Sichuan University, Chengdu, China

**Keywords:** fibroblast, inflammation, keratinocyte, melanocyte, OPN3, opsin 3, photobiology, skin

## Abstract

Opsins are a class of classic G-protein-coupled receptor superfamily members, traditionally known to play a central role in phototransduction in the retina. Recent studies have revealed that various opsins are widely expressed in the skin, making the skin an important peripheral light-sensing organ. Among these, opsin 3 (OPN3), as one of the most abundantly expressed and functionally diverse photosensitive proteins in mammalian skin, has attracted increasing attention. This narrative review systematically outlines the expression profile of OPN3 among photoreceptors in the skin. Building on this, it provides a detailed analysis of OPN3’s molecular structural characteristics, the mechanisms by which it detects light signals (particularly ultraviolet and short-wavelength visible light), and the downstream signal pathways it triggers in various skin cell types (such as keratinocytes, melanocytes and fibroblasts). These pathways include, but are not limited to, the G protein-mediated PLC-IP3/DAG-Ca^2+^ pathway, the cAMP-MITF pathway, and interactions with cytochrome C and the TRP ion channel; together, they regulate important physiological processes in the skin, such as pigment metabolism, immune regulation, barrier function, cell proliferation and differentiation, DNA damage repair, and circadian rhythm synchronization. Furthermore, this paper explores the role of dysregulation in the OP3 signal network in skin pathophysiology, such as its potential role in UV damage, the development of melanoma, photoaging, and inflammatory skin diseases. Finally, this paper outlines the translational prospects of OPN3 as a novel therapeutic target in the treatment of photodermatoses, skin regenerative medicine, and the development of circadian rhythm-modulating skincare products.

## 1 Introduction

As the body’s largest organ, the skin is continuously exposed to ambient light, particularly solar radiation (1, 2). In addition to known UV receptors (such as DNA and porphyrins) (3, 4), a series of light-sensitive proteins responsible for vision in the retina, including members of the opsin family, have been found to be functionally expressed in various skin cells (5). Currently identified non-visual opsins that are functionally expressed in human skin mainly include opsin 3 (OPN3, encephalopsin), opsin 4 (OPN4, melanopsin), and opsin 5 (OPN5, neuropsin). Although OPN1 and OPN2 primarily function as retinal photopigments, emerging evidence suggests that OPN3, OPN4, and OPN5 play functional roles in peripheral tissues, including the skin (1, 6). These enable skin cells to directly detect light of specific wavelengths and initiate rapid intracellular signal cascades, independent of the visual system (7). Among the numerous opsin proteins expressed in the skin, opsin 3 stands out due to its high expression levels (8) and widespread distribution, In the epidermis, it is highly expressed in keratinocytes and melanocytes; in the dermis, expression is observed in fibroblasts, endothelial cells, hair follicle cells, and even detected in mast cells and Langerhans cells (9–12), and its broad spectral response range, capable of responding to wavelengths from the near-ultraviolet to the blue light spectrum, making it particularly noteworthy (13). Consequently, OPN3 is considered one of the core molecules mediating direct photobiological effects in the skin and serves as a key bridge linking environmental light signals to the skin’s physiological and pathological states. Dysregulation of OPN3 signaling has been implicated in a spectrum of dermatological conditions, including ultraviolet (UV)-induced damage, melanoma progression, photoaging, and inflammatory disorders such as atopic dermatitis. Therefore, review aims to comprehensively outline the expression characteristics of OPN3 in the skin, its light-sensing mechanisms, and downstream signaling networks. It systematically summarizes its roles in physiological and pathological processes such as pigmentation regulation, inflammation, photoaging, and angiogenesis, while exploring its translational potential as a therapeutic target.

## 2 Methods

This is a narrative review aimed at providing a comprehensive overview of the multifaceted roles of OPN3 in cutaneous physiology and pathology. We conducted a comprehensive literature search in PubMed, Web of Science, and Scopus up to January 2026, using keywords including “Opsin 3,” “OPN3,” “skin,” “photobiology,” “melanocyte,” “keratinocyte,” “fibroblast,” and “inflammation.” We included original research articles, review papers, and relevant conference proceedings focusing on OPN3 expression, signaling, and functional outcomes. Given the heterogeneity of study designs, we did not perform a meta-analysis or assess risk of bias quantitatively. Instead, we synthesized findings from *in vitro* cellular models, *in vivo* animal models, and human clinical or ex vivo tissue studies. To improve clarity, the results are presented separately by model type where appropriate, and comparisons are drawn to highlight translational relevance.

## 3 Structural and biochemical characteristics of OPN3

OPN3 was identified in the mouse genome in 1999 (14), and shortly thereafter it was found that OPN3 is expressed in a variety of peripheral tissues, including the brain, placenta, retina, liver, heart, lungs, skeletal muscle, pancreas and skin (1, 15). It belongs to the Class A family of G protein-coupled receptors (GPCRs) and possesses a typical seven-transmembrane domain structure (16). Like all rhodopsin, its functional activity depends on covalent binding to a chromophore (chromophore group), typically 11-cis-retinal (derived from vitamin A) (17). The chromophore is linked via a Schiff base bond to a conserved lysine residue on the seventh transmembrane helix, forming a functional opsin-chromophore complex (14, 16). Absorption of photons causes 11-cis-retinal aldehyde to photoisomerise into the all-trans configuration, thereby triggering a dramatic conformational change in the opsin and activating downstream G-protein-coupled signaling (18). A distinctive feature of OPN3 is that the maximum of its absorption spectrum (λ_max_ ≈ 465 nm) typically lies in the near-ultraviolet and blue light regions (approximately 400–500 nm), with the exact position varying slightly depending on the species and tissue environment (13). This spectral characteristic makes it a potential candidate receptor for sensing the UVA (320–400 nm) and blue light (400–495 nm) components of sunlight. Compared with OPN4 (λ_max_ ≈ 490 nm), which is primarily responsible for the photoregulation of circadian rhythms (19, 20), OPN3’s activation spectrum is shifted toward shorter wavelengths, suggesting a unique role in biological processes triggered by higher-energy photons (21). Furthermore, there is evidence suggesting that under certain *in vitro* culture conditions, OPN3 can apparently be activated even without exogenous retinal supplementation. Some researchers propose that serum components in the culture medium may provide sufficient retinoids to form functional chromophores *in situ*, thereby conferring photosensitivity (22).

## 4 The role of OPN3 in human skin

### 4.1 The bidirectional regulatory role of OPN3 in skin pigmentation

Skin pigmentation is a complex physiological process that primarily depends on the synthesis, transport and distribution of melanin within melanocytes (23). Recent studies have revealed that OPN3 plays a key role in the regulation of skin pigmentation and exhibits unique bidirectional regulatory characteristics, both light-dependent and light-independent (24). High-energy visible light, particularly blue light in the 415 nm wavelength band, can activate OPN3, thereby inducing a series of intracellular signal events (25). Studies have shown that when skin melanocytes are exposed to high-intensity blue light (≥50 J/cm^2^), OPN3 is activated and increases cytoplasmic calcium ion flux, thereby activating calcium/calmodulin-dependent protein kinase II (CaMKII), which subsequently phosphorylates cyclic adenosine monophosphate response element-binding protein (CREB), extracellular signal-regulated kinase 1/2 (ERK1/2),mitogen-activated protein kinase (MAPK) p38 and myo-eye-related transcription factor (MITF) (25). As a key regulator of melanocyte development and function, the activation of MITF leads to the upregulation of various melanin-related genes, including tyrosinase (TYR) and tyrosinase-related proteins (TYRP-1 and DCT) (26). Furthermore, blue light activation of OPN3 can induce the formation of multimeric tyrosinase complexes, comprising tyrosinase (TYR) and tyrosinase-related proteins TYRP-1 and TYRP-2 (25, 27). These complexes are stably present on the melanosome membrane and may continuously promote the synthesis of eumelanin by enhancing the activity and stability of tyrosinase (25, 28). This mechanism is particularly pronounced in Fitzpatrick skin types III–VI, explaining why darker skin types exhibit more persistent and pronounced pigmentation following exposure to visible light (25).

**Figure 1:**
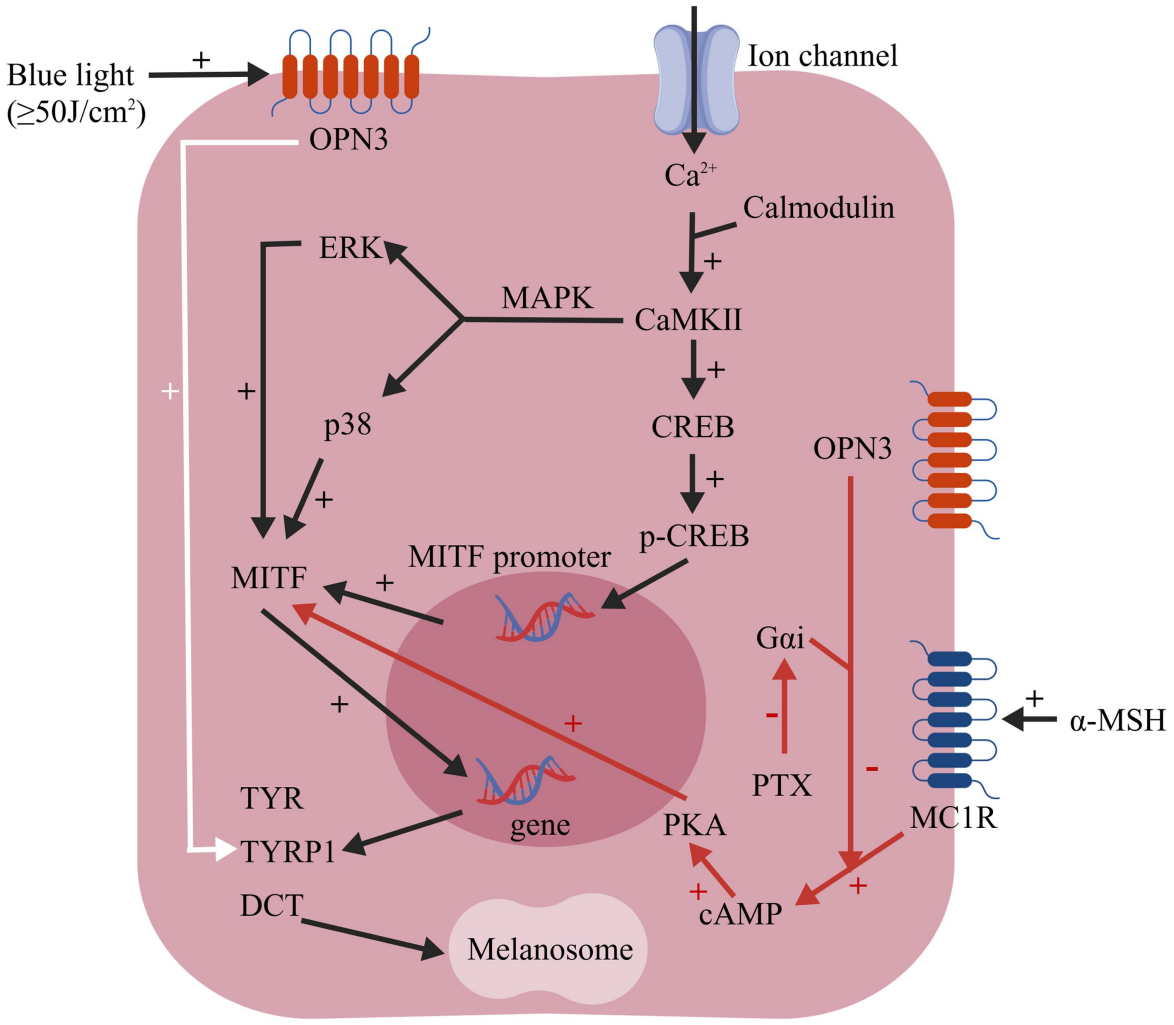
Mechanism of the bidirectional regulatory pathway of OPN3 in skin pigmentation. Created with BioGDP.com.

Surprisingly, the Brown University research team discovered that OPN3 is also involved in a non-light-dependent mechanism regulating pigmentation (29). After using genetic engineering techniques to reduce OPN3 expression in melanocytes, they observed a significant increase in basal melanin content, suggesting that OPN3 may suppress melanin production under normal conditions (29). Further mechanistic studies revealed a functional interaction between OPN3 and the melanocortin 1 receptor (MC1R) (29). MC1R is a key receptor regulating skin pigmentation; when activated by *α*-MSH, it activates adenylate cyclase (cAMP) via the Gαs subunit, increasing intracellular cAMP levels, thereby promoting MITF expression and melanin synthesis (29). OPN3 may inhibit the MC1R-mediated cAMP signal pathway by coupling with Gαi protein, thereby negatively regulating melanin production (30). Evidence supporting this mechanism is that treatment with pertussis toxin (PTX, a Gαi inhibitor) abolishes the inhibitory effect of OPN3 on MC1R-activated cAMP signal (29). This light-independent regulatory mechanism suggests that light dosage may influence OPN3 expression and melanogenesis, indicating the need for further research to determine whether OPN3 maintains the homeostatic balance of skin pigmentation under basal conditions while switching to a protective, pigment-promoting response during high-intensity light exposure (25, 29) (Figure 1).

### 4.2 The role of OPN3 in skin inflammatory responses

Recent studies have revealed that OPN3 not only participates in the regulation of skin pigmentation but also plays a significant role in skin inflammatory responses, particularly in the light accentuation phenomenon of atopic dermatitis (AD) (31). Clinical observations indicate that approximately 3% of AD patients experience exacerbation of symptoms following light exposure, with rashes predominantly distributed on the face, neck, exposed trunk, and light-exposed areas such as the hands and arms (32, 33). This phenomenon has prompted researchers to investigate the role of photoreceptors in the pathogenesis of AD. In 2025, a study by Professor Li Wei’s team at Huashan Hospital, affiliated with Fudan University, found that OPN3 expression was significantly increased in keratinocytes within the lesional areas of AD patients and MC903-induced AD mouse models, whereas no similar changes were observed in psoriatic lesions (31). Further studies have shown that type 2 inflammatory cytokines (such as IL-4 and IL-33) can induce upregulation of OPN3 expression in keratinocytes, providing important clues for understanding the mechanism underlying increased photosensitivity in AD patients (31). Mechanistic studies indicate that visible white light (400–700 nm, 90 mW/cm^2^) promotes the production of pro-inflammatory factors such as IL-36γ, TNF-*α*, IL-1β and IL-23α in keratinocytes via OPN3-mediated signal pathways. In keratinocytes, light-activated OPN3 upregulates the expression of pro-inflammatory factors such as IL-36γ, TNF-α, IL-8 and IL-1β by inducing calcium influx and activating the sphingosine-1-phosphate (S1P)-S1P receptor (S1PR) signal pathway (31). These factors collectively constitute an inflammatory microenvironment, attracting immune cell infiltration and amplifying the inflammatory response. It is worth noting that the pro-inflammatory effects of OPN3 in keratinocytes are microenvironment-specific; it does not directly induce the expression of type 2 inflammatory factors, but primarily promotes the production of inflammatory factors associated with the IL-17 and TNF pathways. This may explain why the light-aggravated phenomenon is more pronounced in AD, which exhibits a mixed inflammatory pattern, whereas it is not evident in psoriasis, which is dominated by type 1 and type 17 inflammation (31). Furthermore, studies have shown that white LED irradiation exacerbates skin inflammation in mouse models of AD but has no significant effect on mouse models of psoriasis, further supporting the disease-specific nature of OPN3’s action (31). These findings not only reveal a new function of OPN3 in skin inflammation but also provide a molecular mechanism explaining how visible light exacerbates AD, offering new targets for the development of therapeutic strategies for light-aggravated AD. In addition to AD, the pro-inflammatory mechanisms mediated by OPN3 may also be involved in other skin pathological processes. For example, the activation of the OPN3 signaling pathway is closely associated with neurogenic inflammation and may exacerbate erythema and burning symptoms in vascular inflammatory diseases such as rosacea. Furthermore, the expression of OPN3 in mast cells suggests its potential involvement in the photo-induced exacerbation of allergic dermatitis and urticaria.

It is well known that acute inflammation is the body’s defensive response to stimuli, and its five classic signs are redness, swelling, heat, pain and functional impairment (34). TRPV1 is an important member of the TRP channel family; it is a non-selective cation channel primarily expressed at the terminals of nociceptive sensory neurons (35), while also being expressed in non-neuronal cells such as keratinocytes and mast cells (36). Its activation leads to the influx of Ca^2+^ and Na^+^, triggering neuronal depolarisation and generating pain signals (37). Concurrently, the influx of Ca^2+^ triggers the release of neuropeptides from sensory nerve endings, such as substance P (SP) and calcitonin gene-related peptide (CGRP), initiating cutaneous neurogenic inflammation (CNI), which induces cutaneous vasodilation and plasma exudation, subsequently leading to erythema, oedema, burning sensation and pain (38, 39). Previous studies have demonstrated the potential mechanism by which OPN3, upon exposure to blue light, activates the TRPV1 signal pathway via G protein-coupled receptors (40). Currently, the mechanism by which OPN3 regulates calcium ion responses remains unclear, but it may be related to its coupling with Gai/o subunits as a GPCR. Upon activation, the Gai/o subunits dissociate, inhibiting cAMP and reducing cAMP accumulation. Meanwhile, the released Gβγ subunits activate phospholipase Cβ (PLCβ). The activated PLCβ rapidly hydrolyzes phosphatidylinositol 4,5-bisphosphate (PIP2) in the inner cell membrane, generating two important second messengers: inositol trisphosphate (IP3) and diacylglycerol (DAG). IP3 triggers calcium ion release from the endoplasmic reticulum, while DAG, through direct or indirect interactions with IP3, increases intracellular calcium concentration (41–43). Experimental evidence indicates that blue light irradiation upregulates the mRNA expression of OPN3 and TRPV1, and that both the phosphorylation and total protein levels of TRPV1 increase, a process that is dependent on OPN3 activity. Treatment with PTX or OPN3 shRNA significantly attenuates blue light-induced TRPV1 phosphorylation and calcium influx, confirming that OPN3 acts upstream of TRPV1. Furthermore, OPN3 knockdown reduced TRPV1 expression, whereas TRPV1 knockdown had no effect on OPN3, further supporting the regulatory role of OPN3 over TRPV1 (40). The elucidation of the OPN3-TRPV1 signal pathway has revolutionized our understanding of skin photobiology and the mechanisms underlying inflammation. It establishes a rapid and efficient transmission pathway from environmental light stimulation to local neurogenic inflammatory responses. In the future, precise regulation of specific components of this pathway is expected to provide novel therapeutic approaches for a range of intractable cutaneous vascular neurogenic inflammatory diseases.

**Figure 2:**
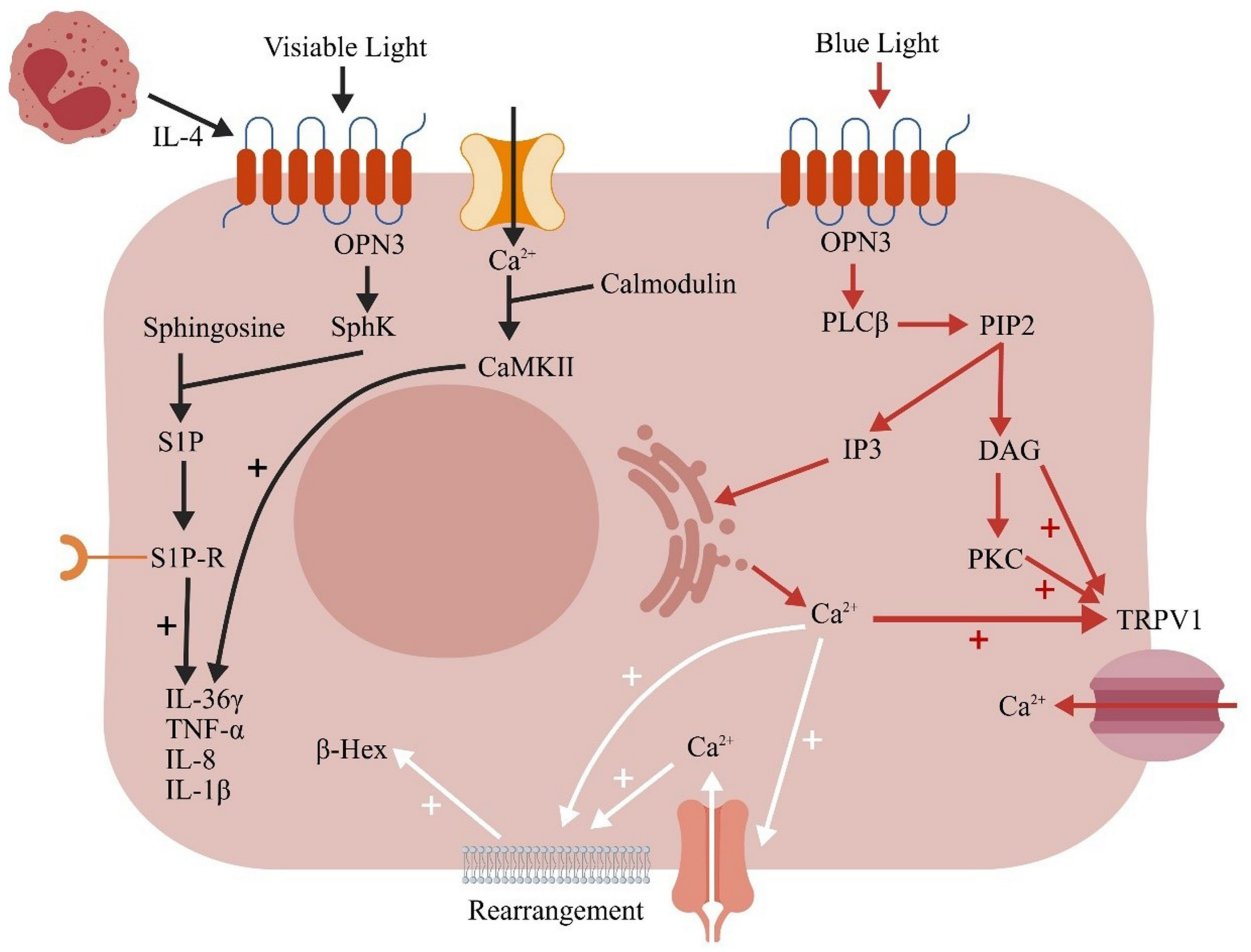
Mechanism of OPN3’s role in the skin’s inflammatory response. Created with BioGDP.com.

Further research indicates that OPN3 is expressed in mast cells as a photoreceptor and mediates blue light-induced degranulation (12). By assessing *β*-hexosaminidase release as a marker of degranulation, this study found that a wavelength of approximately 460 nm significantly induced degranulation; this wavelength corresponds precisely to the absorption peak of OPN3, suggesting a photostimulation effect of OPN3 (12). Mechanistically, OPN3 activation may trigger degranulation by increasing intracellular calcium ion concentrations (44). When OPN3 expression was suppressed via small interfering RNA (siRNA), blue light-induced *β*-hexosaminidase release was significantly reduced, with the effect being most pronounced near 460 nm. Therefore, OPN3 acts as a blue light sensor in mast cells, promoting degranulation via calcium-dependent pathways; this may represent one of the defense mechanisms underlying the inflammatory response of the skin following exposure to intense light (12) (Figure 2).

### 4.3 The role of OPN3 in cutaneous photoaging

**Figure 3:**
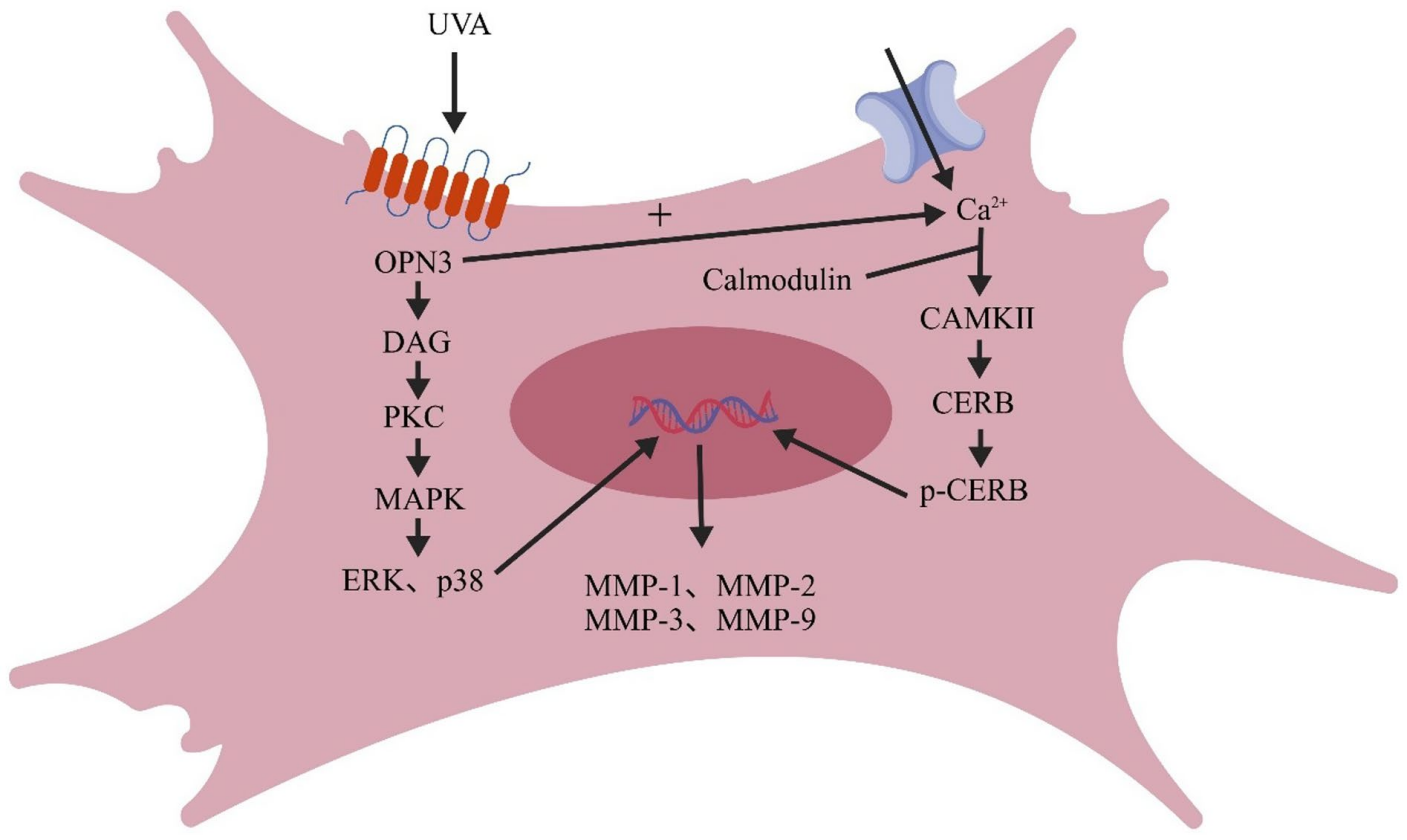
Mechanism of OPN3’s role in skin photoaging. Created with BioGDP.com.

Photoaging is chronic damage to the skin caused by prolonged exposure to ultraviolet and visible light radiation, characterized by wrinkles, loss of elasticity and pigmentation disorders (45). New evidence suggests that ultraviolet A (UVA) can independently induce typical features of photoaging via OPN3 (46). In normal human skin fibroblasts, UVA induces OPN3 expression in a dose-dependent manner, leading to the phosphorylation of CAMKII via calcium-dependent pathways, which in turn induces the phosphorylation of cyclic adenosine monophosphate response element-binding protein (CREB), p38,c-JUN N-terminal kinase (JNK) and extracellular signal-regulated kinase (ERK), significantly upregulating the expression of matrix metalloproteinase-1 (MMP-1), MMP-2, MMP-3 and MMP-9 (46). MMPs are key enzymes in the degradation of core collagen in the skin (47). Prolonged or repeated UVA exposure leads to progressive destruction of the collagen network via this mechanism, resulting in a decrease in skin tensile strength (48, 49). This reveals a new mechanism for understanding UV-induced photoaging, and future research may focus on developing innovative strategies for the prevention and treatment of photoaging by targeting this pathway (Figure 3).

### 4.4 The role of OPN3 in skin angiogenesis

In addition to its functions in melanocytes, keratinocytes and fibroblasts, studies have shown that OPN3 also plays an important biological role in human skin microvascular endothelial cells (50). Functional studies indicate that OPN3 promotes the proliferation, migration and tubule formation of skin microvascular endothelial cells, all of which are key steps in angiogenesis (50, 51). When OPN3 expression was silenced using siRNA technology, these endothelial cell functions were significantly impaired. Mechanistically, OPN3 exerts its effects by forming a complex with vascular endothelial growth factor receptor 2 (VEGFR2) to regulate the activity of key angiogenic signal molecules such as phosphoinositide 3-kinase/protein kinase B (PI3K-AKT) (50). The study also noted that in human umbilical vein endothelial cells (HUVECs) exposed to blue light, OPN3 expression increased and VEGFR2 expression was up-regulated, suggesting that blue light may exert a pro-angiogenic effect via the OPN3-VEGFR2 pathway (50). These findings suggest that OPN3 may be a potential therapeutic target for skin vascular proliferative diseases (such as infantile hemangioma and pyogenic granuloma) and chronic wound healing disorders. However, related research is still in its early stages, and the specific mechanisms require further exploration.

### 4.5 The role of OPN3 in the proliferation, differentiation, and survival of various skin cell types

In human skin, OPN3 is involved in regulating cell proliferation, differentiation and apoptosis, and is essential for maintaining epidermal homeostasis. In keratinocytes, the study by Castellano-Pellicena et al. found that OPN3 is involved in regulating cell differentiation. Using RNA interference (RNAi) to knock down OPN3 eliminated the light-mediated early differentiation of keratinocytes, suggesting that OPN3 may play a role in wound closure (52). The mechanism may involve OPN3 activating the downstream CREB and Ras signaling pathways through cAMP and calcium ion signaling (53). The observation that knocking down OPN3 blocks calcium influx and phosphorylation of CREB and Ca^2+^/calmodulin-dependent protein kinase II (CAMKII) in both non-irradiated and blue light-irradiated human melanocytes supports this hypothesis, though further research is needed (25). In hair follicle cells, Buscone et al.’s study suggests that low-intensity blue light may have a positive effect on hair growth *in vitro* through its interaction with OPN3. Studies have shown that silencing OPN3 in hair follicle outer root sheath cells can lead to changes in the expression of genes controlling proliferation and apoptosis, eliminating the stimulatory effect of blue light on the proliferation of outer root sheath cells. For example, the expression of UL16 binding protein 1 (ULBP1) and p21-activated kinase (PAK2) in hair follicle outer root sheath cells was downregulated, while the expression of sideroflexin 1 (SFXN1) and caveolin 2 (CAV2) was upregulated (10). In melanocytes, the study by Yu Wang et al. found that OPN3 regulates melanocyte survival through calcium-dependent G protein-coupled signaling and mitochondrial pathways (54). The study reveals that OPN3 knockdown reducing intracellular calcium levels and BAD phosphorylation, thereby inducing a decrease in mitochondrial membrane potential, activation of BAX and inhibition of BCL-2. This leads to the disruption of mitochondrial membrane integrity, followed by the release of apoptotic inducers such as cytochrome C from within the mitochondria, ultimately resulting in cell death via the mitochondrial apoptotic pathway (54, 55). In Langerhans cells, the study by Teng Ye et al. revealed that OPN3 regulates the proliferation, cell cycle, and migration of Langerhans cells (11). These functions are associated with the induction of the MAPK signal pathway. Additionally, in fibroblasts, research by Ting Liu et al. demonstrated that the downregulation of the OPN3 gene simultaneously induces ferroptosis, apoptosis, and pyroptosis in human dermal fibroblasts *in vitro* (56). These studies reveal how OPN3 participates in maintaining epidermal homeostasis, promoting wound healing and influencing hair follicle function by regulating skin cells, thereby laying an important foundation for future in-depth exploration of the molecular mechanisms of OPN3 in skin physiology and pathophysiology, as well as the development of related therapeutic strategies.

## 5 OPN3 signal dysregulation and the pathophysiological mechanisms of skin disorders

Abnormalities in the OPN3 signal network are associated with various dermatopathological conditions. In UV-induced skin damage, OPN3 acts as a key sensor for the formation of the melanin cap induced by UVA (57). The melanin cap forms a cover over the nuclei of keratinocytes, acting as a natural sunscreen that protects DNA by absorbing and scattering UV radiation (58, 59). When OPN3 expression is reduced, UV-induced DNA damage increases, may increase the risk of light-induced cancer (57, 60). In melanoma, studies suggest that abnormal OPN3 expression may be associated with tumor cell proliferation and malignant transformation potential (60, 61). In inflammatory skin diseases such as atopic dermatitis, disruption of OPN3 signal in keratinocytes may exacerbate the inflammatory response (31). Furthermore, OPN3-mediated disruption of light signal may disrupt the local skin circadian rhythm, reducing resistance to environmental stressors (62, 63). Understanding these pathological mechanisms provides a theoretical basis for interventions targeting the OPN3 pathway (Table 1).

**Table 1:** Summary of the functions and mechanisms of OPN3 in different skin pathophysiological conditions.

Disease/Pathological condition	Main target cells	Key signaling pathways	Functional outcome
Light-induced carcinogenesis	Keratinocytes	Formation of melanin cap/DNA repair	Protection/promotion (depending on OPN3 expression level)
Melanoma	Melanocytes	cAMP/MITF, calcium signaling	Regulation of proliferation and survival
Atopic dermatitis	Keratinocytes	OPN3-S1P-S1PR, IL-36γ	Pro-inflammatory, exacerbates itching and erythema

## 6 Prospects for translational therapy

In-depth elucidation of OPN3 function provides new theoretical perspectives and practical directions for the treatment of skin diseases and health management. In terms of therapeutic strategies, the development of specific agonists or antagonists targeting OPN3 is expected to become a novel intervention for pigmentary disorders (such as vitiligo and hyperpigmentation), achieving therapeutic goals by regulating pathways related to melanin production. Furthermore, in the prevention of skin cancer, appropriate regulation of OPN3 signal may help enhance the defense capacity of skin cells against UV damage and promote the clearance of cells undergoing early malignant transformation. In phototherapy, further clarification of the spectral range and dose–response relationship of OPN3 could drive the precise optimization of existing narrow-band UVB, UVA and blue light therapies, thereby improving therapeutic efficacy and reducing adverse reactions. Based on OPN3-mediated light signal regulation, exposure to specific wavelengths of light may promote keratinocyte migration, vascular endothelial cell proliferation and hair follicle cell activation, offering novel photobiomodulation approaches for chronic wound healing, scar management and hair regrowth. Furthermore, by leveraging OPN3’s role in the light-synchronization of the skin’s circadian rhythm, it is possible to explore the development of topical circadian rhythm-regulating formulations or design phototherapeutic skincare products with specific light parameters. This would help address skin imbalances caused by disrupted sleep patterns and open up new avenues for daily skin health management. However, clinical translation in this field still faces knowledge gaps and several challenges. Firstly, most mechanistic studies are based on *in vitro* cell models or mouse models, lacking validation from high-quality human clinical trials. Secondly, OPN3 exhibits both light-dependent and light-independent dual activities, making the development of specific agonists or antagonists that do not interfere with its fundamental physiological functions a major challenge in drug development. Future research should focus on utilizing organoids or humanized mouse models to validate the *in vivo* efficacy of OPN3-targeted therapies, resolving the high-resolution structure of OPN3 in different skin cells to aid drug design, and conducting epidemiological studies to clarify the association between OPN3 gene polymorphisms and susceptibility to skin diseases.

## 7 Conclusion

As a key broad-spectrum photoreceptor in the skin, OPN3 profoundly influences skin homeostasis and pathological processes by activating complex and finely tuned light-dependent and light-independent signal networks. From regulating pigment metabolism, immune responses, and cell proliferation and differentiation to maintaining local circadian rhythms in the skin, the OPN3 signal pathway constitutes the core molecular basis for the skin’s adaptation to the light environment. Although its detailed mechanisms, particularly the specifics of signal transduction in different cellular contexts, require further elucidation, existing research has already fully revealed its immense potential as a novel therapeutic target. Future research should focus on elucidating the precise functional landscape of OPN3 signal in human skin, developing highly selective modulators, and advancing the clinical translation of precision therapies and health management strategies based on the principles of OPN3 photobiology, which will bring revolutionary progress to the field of dermatology.

### 7.1 Limitations

This review has certain limitations. Firstly, as a narrative review, it did not employ systematic evaluation scales to assess the risk of bias in the included literature. Secondly, research on OPN3 is still in its early stages, with many mechanisms validated only in single cell lines or animal models. Significant variations in experimental conditions (e.g., light intensity, duration) across different studies make direct comparison and data aggregation challenging. Additionally, the interaction network between OPN3 and other membrane receptors (such as MC1R and VEGFR2) is highly complex. Current evidence consists mostly of fragmented pathway segments, and there is a lack of integrated systems biology analyses to explain its overall physiological effects *in vivo*. Finally, it should be noted that this review focuses on the expression and function of OPN3 in the skin. However, as another type of opsin, OPN4 shares overlapping absorption spectra with OPN3 and has similar peak wavelengths. Therefore, these two opsins may cooperatively or competitively mediate light signals in the skin, and future studies should evaluate the roles of both receptors simultaneously.
